# Various Hydrogel Types as a Potential In Vitro Angiogenesis Model

**DOI:** 10.3390/gels10120820

**Published:** 2024-12-12

**Authors:** Chloé Radermacher, Annika Rohde, Vytautas Kucikas, Eva Miriam Buhl, Svenja Wein, Danny Jonigk, Willi Jahnen-Dechent, Sabine Neuss

**Affiliations:** 1 Biointerface Laboratory, Helmholtz-Institut for Biomedical Engineering, University Hospital RWTH Aachen, Pauwelsstraße 30, 52074 Aachen, Germanysabineneussstein@me.com (S.N.); 2 Institute for Molecular Cardiovascular Research (IMCAR), RWTH Aachen University, Pauwelsstrasse 30, 52074 Aachen, Germany; 3 Electron Microscopy Facility, University Hospital RWTH Aachen, Pauwelsstrasse 30, 52074 Aachen, Germany; 4 Institute of Pathology, RWTH Aachen University, Pauwelsstrasse 30, 52074 Aachen, Germany; 5 German Center for Lung Research (DZL), Biomedical Research in Endstage & Obstructive Lung Disease (BREATH), Carl-Neuberg-Str. 1, 30625 Hannover, Germany

**Keywords:** angiogenesis, hydrogels, collagen, human platelet lysate, fibrin, co-culture

## Abstract

Angiogenesis, the formation of new blood vessels, is a fundamental process in both physiological repair mechanisms and pathological conditions, including cancer and chronic inflammation. Hydrogels are commonly used as in vitro models to mimic the extracellular matrix (ECM) and support endothelial cell behavior during angiogenesis. Mesenchymal stem cells further augment cell and tissue growth and are therefore widely used in regenerative medicine. Here we examined the combination of distinct hydrogel types—fibrin, collagen, and human platelet lysate (HPL)—on the formation of capillaries in a co-culture system containing human umbilical vein endothelial cells (HUVECs) and bone marrow-derived mesenchymal stem cells (BM-MSCs). The mechanical properties and structural changes of the hydrogels were characterized through scanning electron microscopy (SEM) and nanoindentation over 10 days. Fibrin and HPL gels sustained complex network formations, with HPL gels promoting even vascular tube formation of up to 10-fold capillary caliber. Collagen gels supported negligible angiogenesis. Our results suggest that HPL gels in combination with MSC-EC co-culture may be employed to obtain robust vascularization in tissue engineering. This study provides a comparative analysis of fibrin, collagen, and HPL hydrogels, focusing on their ability to support angiogenesis under identical conditions. Our findings demonstrate the superior performance of HPL gels in promoting robust vascular structures, highlighting their potential as a versatile tool for in vitro angiogenesis modeling.

## 1. Introduction

Angiogenesis, the formation of new blood vessels, is a critical process in both physiological conditions such as tissue repair, and pathological states like cancer and chronic inflammation [1,2,3,4]. In vitro models of angiogenesis provide a valuable platform for investigating the underlying mechanisms and offer a controlled environment for manipulating factors that influence different modes of capillary formation [5,6]. Hydrogels, due to their biocompatibility and ability to simulate the extracellular matrix (ECM), are frequently used in these models to support endothelial cell behavior [7].

Mesenchymal stem cells provide multiple regenerative signaling and are therefore the focus of much research in basic and applied regenerative medicine [8,9]. This study aims to investigate how different hydrogels—fibrin, collagen, and human platelet lysate (HPL)—in combination with co-cultures of endothelial cells and mesenchymal stem cells influence capillary formation, sprouting angiogenesis, intussusceptive angiogenesis, and vessel elongation. Sprouting angiogenesis is the process by which new blood vessels form through the proliferation and migration of endothelial cells from pre-existing vessels, resulting in the creation of capillary branches [10]. This mode of angiogenesis is essential during wound healing and tissue growth, as it facilitates the expansion of the vascular network through new vessel formation [11,12]. Intussusceptive angiogenesis (also known as splitting angiogenesis) involves the division of existing vessels through the formation of intraluminal tissue pillars, allowing for the vessel lumen to split into two without extensive endothelial cell migration or proliferation [13]. This process is crucial for the rapid remodeling and expansion of vascular networks without the need for new vessel sprouting [14,15]. Vessel elongation refers to the lengthening and maturation of existing blood vessels, which play a key role in extending the vascular network [16]. This process does not involve the formation of new branches but rather the growth and stabilization of pre-formed capillaries, contributing to the overall expansion and functionality of the vascular system [17]. While individual hydrogels have been extensively studied, their comparative potential for supporting angiogenesis under the same experimental framework has not been fully explored. This study seeks to address this gap by systematically evaluating whether and to what extent fibrin, collagen, and HPL hydrogels facilitate the formation of vascular structures.

The three types of hydrogels investigated in this study each provide distinct environments that influence endothelial cell behavior and capillary formation. Fibrin gels comprise the fibrous ECM of clotted blood plasma, which is known to support endothelial cell migration and attachment, making them ideal for studying sprouting angiogenesis [18,19]. Collagen gels, due to their close resemblance to the native ECM, promote the formation of interconnected capillary structures and facilitate intussusceptive angiogenesis [20,21,22]. Human platelet lysate (HPL) gels resemble fibrin gels in that they contain fibrin as the major component. In addition, HPL is rich in platelet-derived growth factors and cytokines. This further promotes endothelial cell proliferation and migration, creating an optimal environment for vessel elongation and maturation. HPL-based hydrogels thus form a stable, biodegradable matrix that supports cell adhesion, tissue regeneration, and efficient delivery of bioactive molecules [23,24,25].

Co-cultures of bone marrow-derived mesenchymal stem cells (BM-MSCs) and human umbilical vein endothelial cells (HUVECs) in combination with synthetic hydrogels have been described as a robust model for studying angiogenesis [26,27]. This approach harnesses regenerative properties of both cell types: HUVEC endothelial cells are critical for forming vascular-like networks, and BM-MSCs enhance angiogenesis through paracrine signaling, extracellular matrix remodeling, and differentiation potential [28,29].

By comparing the effects of three hydrogel types—fibrin, collagen, and HPL—this study seeks to elucidate how different matrix environments influence angiogenesis and vascularization in three-dimensional co-culture of HUVECs and MSCs. We aimed to develop a versatile in vitro model for angiogenesis, with potential applications in drug discovery, regenerative medicine, and the study of pathological conditions such as cancer and ischemic diseases.

## 2. Results and Discussion

### 2.1. Cell Characterization

**Figure 1:**
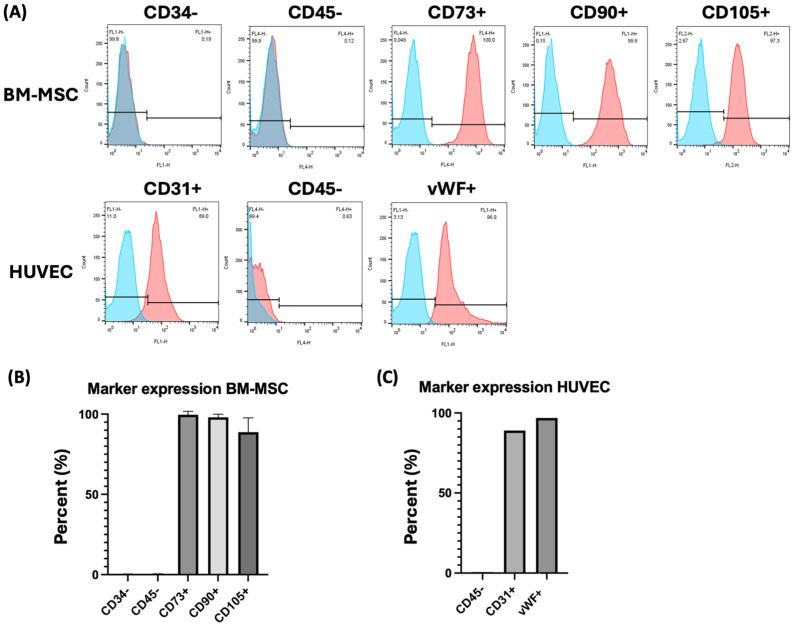
Characterization of MSCs and HUVECs using flow cytometry. (**A**) In the upper row: a typical flow cytometric analysis of one exemplary MSC donor for stem cell markers, which should stain positive (CD73+, CD90+, CD105+), as well as surface markers of hematopoietic stem cells and endothelial cells, which should stain negative (CD34−, CD45−). In the lower row: a typical flow cytometric analysis of the HUVEC donor for endothelial-specific markers (CD31+ and vWF+) and hematopoietic stem cells, which should be stained negative (CD45−). (**B**) Flow cytometric quantification of surface marker expression of the 3 donors of BM-MSCs. CD34− and CD45− were negative for all cell types. High levels of CD73+ (>99.8), CD90+ (>99.8%), and CD105+ (>98.8%) expression were detectable in all stem cell types. (**C**) Flow cytometric quantification of surface marker expression of the three donors of each cell type. CD45− was negative and CD31+ and vWF+ achieved high levels of expression, at 89.1% and 98.25%, respectively. The percentage indicates the percentage of cells that express the respective marker. Abbreviations: BM-MSCs, bone marrow-derived mesenchymal stem cells; HUVECs, human umbilical vein endothelial cells; vWF, von Willebrand factor. Biological *n* = 3 for BM-MSCs and *n* = 1 for HUVECs.

BM-MSCs and HUVECs were primary cells isolated from native patient tissues, and their cellular identity was confirmed by flow cytometry. According to the International Society for Cellular Therapy, BM-MSCs are negative for hematopoietic stem cell and lymphocyte surface markers CD34− and CD45−, respectively, with expression levels lower than 5%, and are positive for CD73+, CD90+, and CD105+. As shown in Figure 1A,B, the mean expression levels were 0.24 ± 0.17% for CD34− and 0.28 ± 0.21% for CD45−, whereas the positive markers showed expression levels of 99.63 ± 0.45% for CD73+, 98.07 ± 1.55% for CD90+, and 88.8 ± 7.29% for CD105+.

Figure 1A,C illustrates the expression on HUVECs of endothelial markers CD31+ and vWF+ and the absence of the lymphocyte antigen CD45− as identification criteria. Primary HUVECs exhibited a high expression of CD31+, at 89%, and the universal endothelial cell marker vWF+, at 96.9%, while being negative for CD45−, with an expression level of 0.63%.

### 2.2. Angiogenesis W/O Added Hydrogel

HUVECs were seeded onto a layer of BM-MSCs to assess the capacity of the selected HUVEC donor to form capillary-like structures. To evaluate the system’s responsiveness to different factors, three experimental conditions were tested: supplementation with VEGF, addition of suramin, or no additives. VEGF, a well-established pro-angiogenic factor, was expected to enhance capillary-like structure formation, while suramin, an angiogenesis inhibitor, was anticipated to suppress this formation.

**Figure 2:**
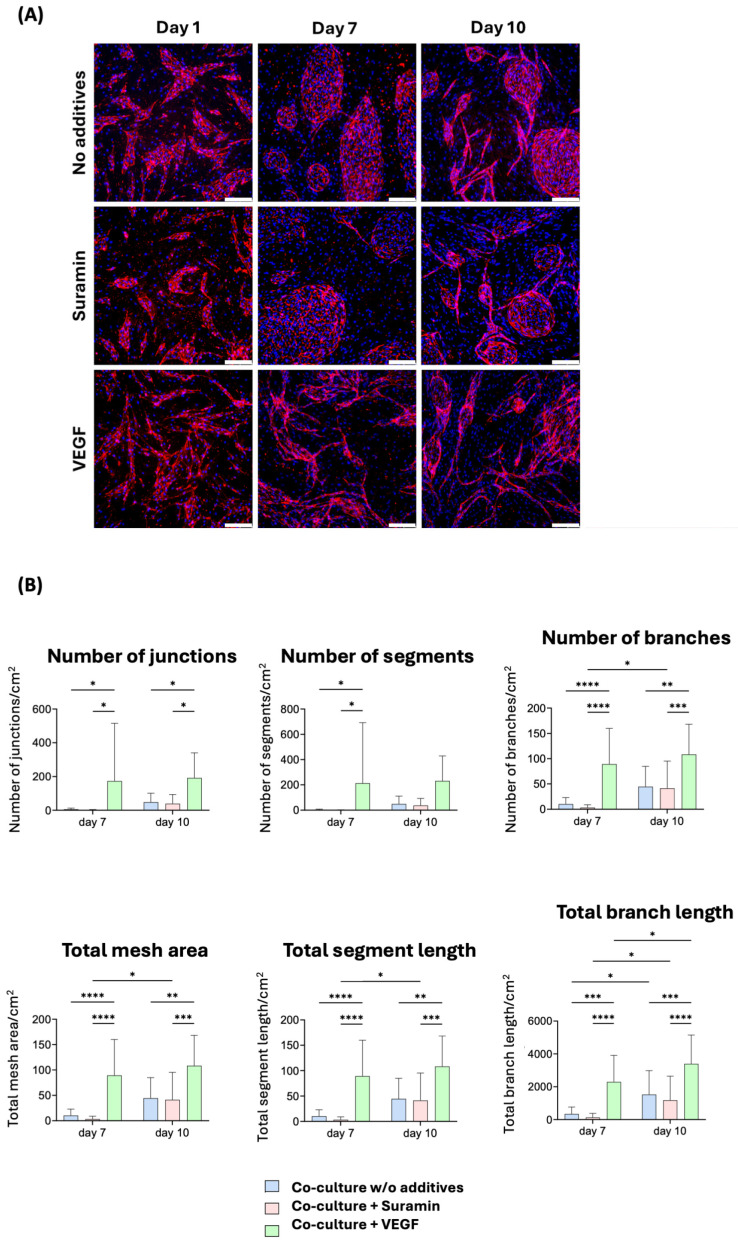
Two-dimensional angiogenesis experiments. (**A**) Representative immunofluorescence-staining pictures with no additive, suramin, or VEGF addition on days 1, 7, and 10. CD31+ is shown in red and cell nuclei in blue (DAPI). Images were taken at 100× magnification; scale bar: 200 μm. (**B**) Quantification of the fluorescent microscopy pictures showing the number of branches/segments/junctions, total branch/segment length, and total mesh area of the three conditions on days 7 and 10. Two-way ANOVA was applied (*p* * ≤ 0.01, *p* ** ≤ 0.005, *p* *** ≤ 0.001, *p* **** ≤ 0.0001) with a Tukey post hoc test.

To monitor tube formation over time, samples were fixed at days 1, 7, and 10 post-seeding. Immunofluorescence images (Figure 2A) demonstrated the presence of capillary-like structures in all conditions by day 10, with initial structure formation observed as early as day 7, particularly in the VEGF-treated samples.

Quantification of these structures was performed using the angiogenesis analyzer plugin for ImageJ [30], as shown in Figure 2B. By day 10, VEGF-treated samples exhibited a significant increase in capillary-like structures compared to the control across all parameters except total branch length. Notably, total branch length in mm per cm^2^ observed area was 8.7-fold greater on day 7 in the VEGF-treated condition (89.3 ± 68.1 /cm^2^) compared to the control (10.3 ± 12.3 /cm^2^). Similarly, the number of branches (n per cm^2^ observed area) was 6.7-fold higher on day 7 with VEGF treatment (2294.4 ± 1558.3 /cm^2^) compared to the control (344.5 ± 408.6 /cm^2^).

Although there were no differences between the control and VEGF-treated conditions in some parameters, significant differences were often observed between the suramin and VEGF conditions. In the control condition, a significant increase in most parameters from day 7 to day 10 was noted, except for mesh size, indicating that the majority of capillary-like structure formation occurred during this period. After 10 days, no significant change was noted with VEGF supplementation, suggesting that these structures may lack long-term stability and that a 10-day experimental window is sufficient for subsequent investigations.

### 2.3. Hydrogel Characterization

**Figure 3:**
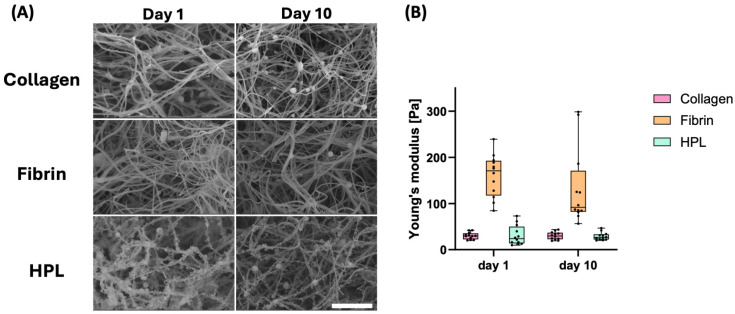
Characterization of the hydrogels. (**A**) SEM pictures of the three different hydrogels after 1 day and 10 days in culture. Magnification of 20,000× with a scale bar of 3 µm. (**B**) Determination of Young’s elastic modulus of the hydrogels using nanoindentation to investigate stiffness and stability over time. Two-way ANOVA was applied (*p* * ≤ 0.01).

The fiber structure of the extracellular matrix (ECM) is well known for its ability to regulate cellular morphology, proliferation, migration, and gene expression, making it a critical parameter in hydrogel characterization. Given that initial experiments indicated no further capillary formation beyond 10 days, subsequent experiments were terminated at that time point. Figure 3A presents SEM images of each hydrogel type after 1 and 10 days of culture without additives, captured at 20,000× magnification.

Hydrogels containing human platelet lysate (HPL) exhibited distinct characteristics compared to the other two hydrogels. Specifically, HPL fibers displayed fringe-like structures rather than a smooth surface. Additionally, HPL fibers appeared to be thinner and more uniform in thickness compared to the fibers in the other hydrogels. Over the 10-day period, visual analysis of SEM images (Figure 3A) revealed consistent fiber morphologies across time points, with no apparent structural changes in any of the hydrogels. Although this analysis is qualitative, it aligns with prior studies demonstrating the stability of fibrin and collagen fibers under similar conditions.

As both fibrin gels and HPL are composed of fibrin fibers, a similar structure was anticipated. However, the absence of additional thrombin in the formation of HPL gels may account for the observed thinner fibers. The presence of other components from the plasma in HPL likely contributed to the fringe-like structures observed.

A distinction between the hydrogels was observed in terms of mechanical properties, shown in Figure 3B. Collagen and HPL hydrogels exhibited a Young’s modulus of approximately 30 Pa, while fibrin hydrogels were stiffer, with a modulus of around 150 Pa. These stiffness values are comparable to those of very soft tissues. Mechanical properties, as represented by Young’s modulus (Figure 3B), showed no discernible trends over the 10-day period. These observations indicate that the physical properties of the hydrogels remained constant throughout the experimental timeline, supporting their suitability as stable scaffolds for angiogenesis experiments.

### 2.4. Angiogenesis in Hydrogels

In collagen hydrogels, after one day in culture, HUVECs maintained a circular morphology and did not exhibit significant spreading across all three experimental conditions. By the end of the first week, prominent tube formation was observed, with the number of tubes continuing to increase up to day 10. The tubes formed within the collagen hydrogels were uniform in size and displayed numerous free endings. Inverted transmitted light microscopy images revealed distinct differences between the conditions after seven days of culture: Samples supplemented with VEGF showed enhanced tube formation, whereas those treated with suramin exhibited reduced tube development and lacked islet formation (Supplementary Data Figure S1–S3). However, by day 10, extensive tube networks were present in all conditions, making differentiation between them challenging. Notably, the tubes were distributed throughout various planes of the hydrogel, indicating evident three-dimensional growth behavior.

**Figure 4:**
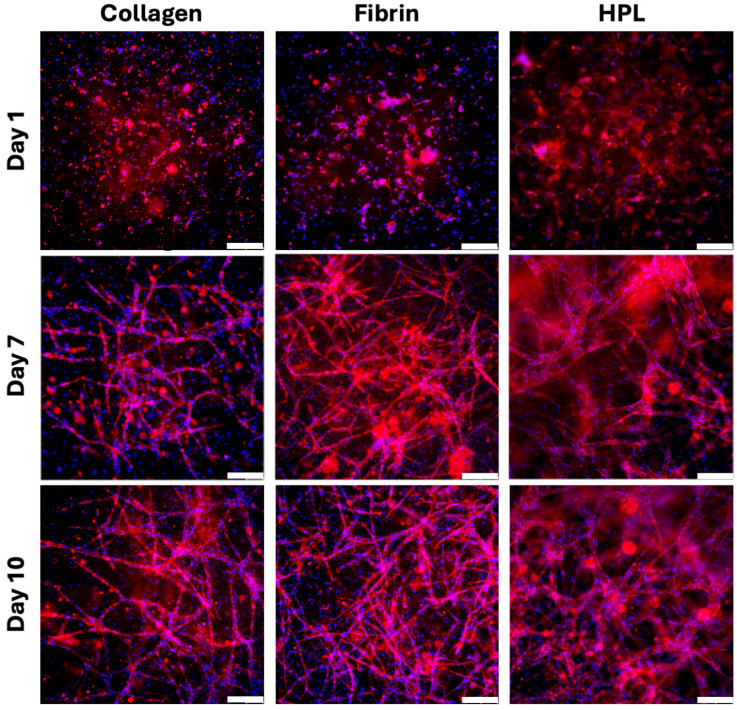
Angiogenesis in hydrogels—representative immunofluorescence pictures of collagen, fibrin, and HPL on days 1, 7, and 10. Experiments were repeated with three different MSC donors. CD31 is shown in red and cell nuclei in blue (DAPI). Images were taken at 100× magnification; scale bar: 200 μm.

In fibrin hydrogels, similar but more pronounced structural developments were detected compared to collagen hydrogels. After one day, HUVECs had noticeably spread out in all conditions. Within a week, robust tubular structures had formed and persisted through day 10. Qualitative analysis of tube morphology suggests fewer free endings in fibrin and HPL gels compared to collagen (Figure 4). This trend, while not quantitatively assessed, is visually evident in the network density and interconnectedness across conditions. The differential effects of supplementation were most apparent on day 7, with suramin-treated samples displaying fewer structures and VEGF-supplemented samples exhibiting increased tube formation. By day 10, extensive and elaborate tube networks were evident across all conditions.

HUVECs co-cultured within human platelet lysate (HPL) gels exhibited distinct behavior compared to the other hydrogel types. On day 1, the cells were extensively spread out, suggesting a highly supportive environment for their proliferation and differentiation. Complex networks comprising tubes of varying sizes were predominantly observed near the edges of the wells. This extensive tube formation was effectively visualized as illustrated in Figure 4. Due to the intensity and complexity of the structures, clear distinctions between days 7 and 10, as well as between different supplementation conditions, were not discernible without quantitative analysis. While quantitative assessments would strengthen these findings, the study focused on establishing qualitative trends to guide future work in this area.

Collectively, these observations indicate that extensive tube formation occurred throughout the entire well across all hydrogel types during the angiogenesis experiments with hydrogels. Collagen hydrogels facilitated the formation of the least complex and dense structures, while fibrin gels supported a more extensive and homogeneous network development. HPL gels promoted the formation of highly complex and heterogeneous angiogenic structures, indicating that HPL best mimics a wound clot environment, which is naturally strongly conducive of angiogenesis. HPL hydrogels exhibited the most complex and extensive network of vascular structures, confirming their superior support for angiogenesis in comparison to fibrin and collagen hydrogels. The co-culture system in HPL led to the formation of larger vessel calibers with fully formed lumens, particularly at the periphery, where vessel formation was most abundant. The substantial vessel elongation observed in the HPL condition highlights its capacity to support the maturation and extension of existing blood vessels. Moreover, the intussusceptive angiogenesis (also termed splitting angiogenesis) observed here played a critical role in the rapid expansion of these complex networks without relying on extensive endothelial cell migration. This outcome indicates that the combination of HPL and co-culture conditions creates an optimal environment for producing large and mature vascular structures.

**Figure 5:**
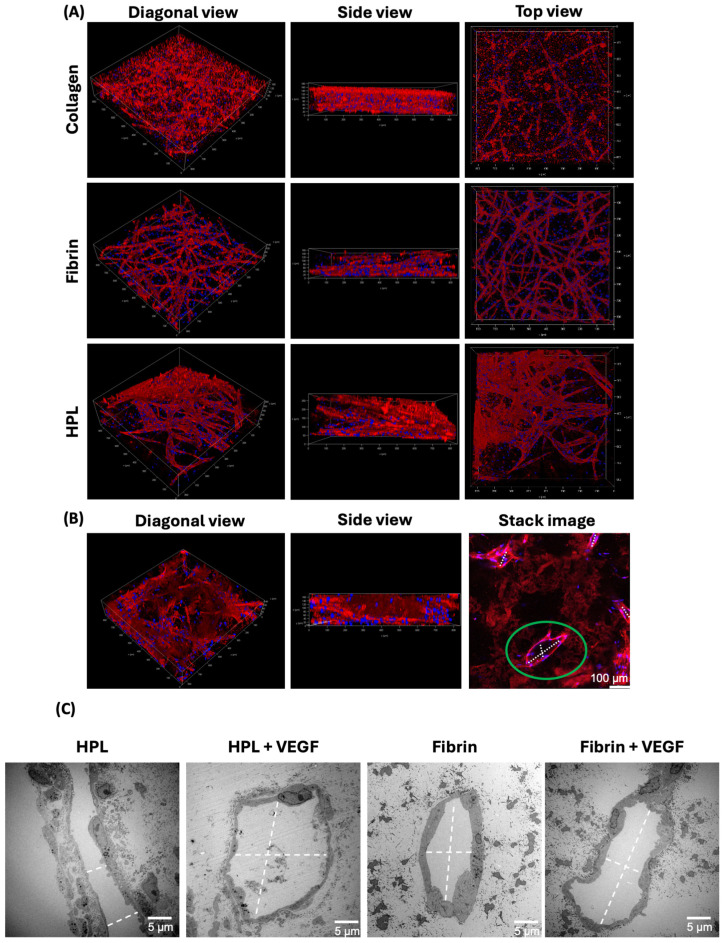
Angiogenesis in hydrogels—2-photon microscopy and TEM. (**A**) Immunofluorescence staining with no additive for each of the three gel types (fibrin, collagen, and HPL). For each gel type the diagonal, side, and top views are shown. (**B**) Diagonal and side views and stack image of HPL gel. The green circle highlights a formed lumen. CD31 is shown in red and DAPI in blue. The diagonal view, side view, and one stack image are shown for an immunofluorescence staining. (**C**) TEM visualization of lumens in HPL and fibrin gels with and without VEGF. The white dashed line indicates the lumen diameter for quantification.

Two-photon microscopy provided 3D visualization of the cellular and tubular architectures across different hydrogel types without supplementation, as presented in Figure 5A,B. In collagen hydrogels, short, unconnected tubes were observed from the top view, with DAPI-stained nuclei distributed across various planes, indicating some degree of 3D arrangement. Fibrin hydrogels supported extensive network formation of micrometer-sized capillaries, mostly localized near the bottom layer but some extending vertically through the gel. HPL gels supported the formation of intricate networks, and additional imaging confirmed that these tubes possessed larger-caliber, closed lumens. Figure 5C illustrates TEM analysis, showing the presence of large-caliber, closed lumens in the vascular structures formed within both HPL and fibrin hydrogels, with and without VEGF supplementation. The TEM images corroborate the two-photon microscopy observations, demonstrating that the lumens were indeed enclosed, which provides further evidence for robust angiogenesis and, indeed, vasculogenesis of EC-MSC co-cultures in HPL gels. In contrast to fibrin hydrogels, where the lumens remained small and poorly organized despite VEGF supplementation, HPL hydrogels demonstrated the formation of much larger and more mature vessels, with a lumen diameter of up to 193.75 μm. This pronounced difference in lumen size highlights the ability of HPL to support the development of well-defined, functional vascular structures, far surpassing the narrow and irregular lumens observed in fibrin (e.g., maximally 20.33 μm without VEGF and 25.63 μm with VEGF). So, the vascular structures observed in HPL gels consistently exhibited larger calibers and more pronounced lumens compared to other hydrogels, as seen in Figure 4 and Figure 5. While these findings are qualitative, they offer a meaningful comparison of hydrogel performance in supporting angiogenesis. Moreover, two-photon microscopy did not reveal comparable levels of vessel maturation in fibrin hydrogels, underscoring the strong potential of HPL to drive the formation of closed, functional vascular networks that more closely resemble physiological capillary structures.

### 2.5. Discussion

Human platelet lysate (HPL) has been known for several years now to promote mesenchymal stem cell expansion, offering a welcome alternative to ill-defined fetal bovine serum as a growth-promoting agent [31]. More recently, HPL was found to stimulate microvascular network formation by endothelial progenitors as well [24].

The present study explored the impact on angiogenesis in MSC-EC co-culture of three different hydrogels: fibrin, collagen, and human platelet lysate (HPL). The fibrin and HPL systems, through their distinct matrix compositions, were able to support endothelial cell organization into angiogenic networks. HPL gels fostered the most extensive and complex angiogenic networks, characterized by vessel formation with up to a 10-fold increase in functional lumen size compared to fibrin. This may be attributed to HPL’s potential to support both intussusceptive angiogenesis, which enables rapid remodeling of vessels without extensive endothelial migration, and vessel elongation, which contributes to the maturation and stabilization of pre-existing vessels. These processes are crucial for developing well-organized, functional vascular structures that resemble physiological conditions [4]. In contrast, fibrin gels supported smaller, less organized lumens even when supplemented with VEGF, and collagen gels exhibited minimal angiogenesis. These findings underscore HPL’s potential as a superior hydrogel for in vitro angiogenesis modeling and tissue engineering applications, particularly in settings where robust and mature vascularization is required.

Under the same conditions with only HUVEC cells, no such structures are formed. Although the terms 2D and 3D co-culture angiogenesis are often used to describe these models, this distinction was deliberately avoided. Even when cultured in well plates, angiogenesis cannot be accurately described as 2D functional vasculature. Instead, the study focused on the conditions with or without hydrogel, as a more appropriate representation of the system’s complexity.

The fibrin hydrogel results corroborate previous reports that fibrin gels facilitate endothelial cell attachment and migration, promoting sprouting angiogenesis. Similar patterns of network formation in fibrin matrices, supporting its established role in angiogenic studies, are already published [18,32]. However, the collagen gels in this study showed minimal angiogenic activity. This contrasts with previous findings [22,33] reporting collagen as a robust matrix for endothelial network formation. The limited angiogenesis observed in collagen gels here may be attributed to differences in collagen concentration, matrix preparation, or cell seeding density, all influencing gel properties [34,35,36].

The HPL gels exhibited the most favorable environment for angiogenesis, supporting the development of complex, non-homogeneous tube networks with closed lumens. This finding agrees with several studies [37,38], where HPL’s high concentration of growth factors (e.g., VEGF, PDGF) was shown to enhance endothelial cell proliferation and that these growth factors also support tube formation. The results suggest that HPL not only supports endothelial cell migration but also promotes lumen formation, making it a promising candidate for in vitro angiogenesis modeling. However, the undefined composition of HPL remains a limitation, as variations in growth factor concentrations could contribute to inconsistencies across experiments.

In addition, the impact of suramin treatment on angiogenesis was also evaluated within this study. Fluorescence data included in Supplementary Figures S1–S3 show a marked reduction in angiogenesis by day 7 under suramin treatment. However, by day 10, angiogenic networks exhibited partial recovery, indicating dynamic changes in the co-culture system. This recovery may be attributed to compensatory mechanisms where mesenchymal stem cells (MSCs) and HUVECs counteracted suramin’s inhibitory effects over time. These findings highlight the resilience of the MSC-EC co-culture model and underscore the need for further research into the molecular pathways involved in these compensatory processes.

The differences in angiogenic behavior across the three hydrogels can be explained by their structural and biochemical properties. Fibrin, known for its fibrous ECM-like structure, provides a scaffold that mimics the native environment, facilitating endothelial sprouting [18]. HPL, rich in growth factors, likely enhanced endothelial proliferation and migration, leading to more complex network formations [24]. In contrast, collagen’s limited ability to promote 3D network formation may be due to its structure, which could hinder cell motility and sprouting [36]. While maximum values for tube diameter are reported, a detailed statistical analysis with means, standard deviations, and comparisons between hydrogels was not performed. Future work should incorporate such quantitative assessments to provide a more comprehensive evaluation of the differences between conditions. This would allow for statistically robust claims about the angiogenic potential of each hydrogel type.

The physicochemical properties of hydrogels, particularly their stiffness, porosity, and biochemical composition, are critical determinants of their impact on angiogenesis. Matrix stiffness, for example, directly influences cell behavior by modulating mechanotransduction pathways, which are crucial for endothelial sprouting, migration, and tube formation [39]. Stiffer matrices can promote sprouting angiogenesis by providing the necessary mechanical cues, while overly rigid or soft matrices may impede cell migration and vessel formation [40]. The fibrin hydrogel’s significantly higher Young’s modulus compared to HPL and collagen made it conducive to sprouting angiogenesis but limited its ability to support the extensive lumen formation observed in HPL. This discrepancy may stem from the biochemical composition of HPL, which provides an abundance of pro-angiogenic factors such as VEGF, in addition to its tunable mechanical properties. In contrast, the collagen hydrogel’s comparatively lower stiffness and more homogeneous structure may have limited cell motility and reduced the formation of functional vascular networks. These findings highlight the importance of selecting hydrogels with optimal physicochemical properties for angiogenesis studies and underscore the rationale for exploring HPL as a model system. Its unique ability to combine biochemical support with suitable mechanical properties makes it particularly advantageous for mimicking in vivo-like conditions, where both structural and biochemical cues drive vascular development.

One limitation of this study is the high variability of natural hydrogels like HPL, where batch-to-batch differences in growth factor composition could affect reproducibility. This point has been addressed by a recent position statement from the working party on cellular therapies of the International Society of Blood Transfusion seeking Good Manufacturing Practice (GMP)-compliant manufacturing protocols for HPL as an ancillary material for the mass production of mesenchymal stem cells for cell therapy [41]. These recommendations should be followed when considering HPL for endothelial cell culture and therapy. GMP production and clinical approval of co-culture are critically required for clinical translation. Further research should also focus on optimizing HPL for therapeutic applications and exploring its performance in more complex, in vivo-like environments using animal models.

Overall, HPL shows significant promise as a robust matrix for supporting complex vascular network formation, with potential applications in both angiogenesis modeling and therapeutic tissue engineering.

## 3. Conclusions

This study demonstrates the distinct effects of fibrin, collagen, and HPL hydrogels on angiogenesis, with each hydrogel supporting different levels of endothelial cell behavior and capillary formation. Fibrin gels facilitated the formation of well-connected tubular structures, while collagen gels exhibited limited angiogenic potential. HPL gels, however, promoted the most complex and extensive angiogenic networks, with closed lumens. These findings underscore the potential of HPL gels as superior matrices for in vitro angiogenesis models. Despite these promising results, the variability in HPL composition presents challenges for standardization. To address this, further work should focus on defining and controlling growth factor concentrations within HPL formulations, possibly through purification or recombinant techniques, to enhance reproducibility and consistency across experiments. Future studies should focus on stabilizing HPL gels and further optimizing hydrogel compositions to enhance control over capillary formation processes. This work highlights the unique ability of HPL gels to support the development of vascular networks more effectively than fibrin and collagen under the tested conditions. These findings contribute to the broader understanding of how different hydrogels influence angiogenesis and support their application in tissue engineering and vascular research. Additionally, exploring the use of HPL gels in more complex in vivo-like environments, such as animal models, will be crucial for validating their therapeutic potential and translational application in regenerative medicine and disease modeling. Overall, these hydrogels provide valuable platforms for studying angiogenesis, with implications for drug discovery, regenerative medicine, and the investigation of pathological conditions like cancer and ischemic diseases.

## 4. Materials and Methods

### 4.1. Cell Culture

BM-MSCs were isolated with the informed consent of patients from femoral heads during hip replacement surgery at the Orthopedic Clinic of the University Hospital RWTH Aachen as previously published (*n* = 3) [42,43,44]. The BM-MSC medium was Mesenpan^®^ Basal Medium containing 2% FBS, 1% ITS-plus (insulin, transferrin, selenium, bovine serum albumin, linoleic acid, 1 nM dexamethasone, 100 μM ascorbic-acid-2-phosphate, 10 ng/mL epidermal growth factor (all Pan-Biotech, Mannheim, Germany), 1.6 mM L-glutamine (Gibco, Langenselbold, Germany), 80 IU/mL penicillin (Gibco, Langenselbold, Germany), and 80 μg/mL streptomycin (Gibco, Langenselbold, Germany).

Umbilical cords were collected with informed consent at the delivery room of the University Hospital RWTH Aachen. The isolation protocol of HUVEC was performed as described [33] (*n* = 1) [45]. The EGM-2 culture medium used for the HUVECs contained EBM-2 medium, 2% FBS, 0.1% hEGf, 0.04% hydrocortison, 0.1% VEGF, 4% hFGF-B, 0.1% R3-IGF-1, 0.1% ascorbic acid, 0.1% gentamicin, and 0.1% heparin (all Lonza, Wupperta, Germany).

All cells were cultured in a 5% CO_2_, 20% O_2_ humidified atmosphere at 37 °C. The medium was changed every 3 to 4 days. All experiments were performed with cells of a maximum passage of 3.

### 4.2. Flow Cytometry

Flow cytometry was used to confirm the identity of primary isolated cells. For each flow cytometry sample, cells were trypsinized, counted, and washed once in flow cytometry buffer (0.09% FBS in phosphate-buffered saline (PBS; Gibco, Germany)). After centrifuging at 500 g for 5 min at 4 °C, the cells were resuspended in 100 μL of flow cytometry buffer, to which corresponding antibodies were added. Following this, quantities of APC-, PE-, or FITC-isotype controls were added: 0.2 μg, 0.2 μg, and 0.5 μg (Becton Dickinson, Heidelberg, Germany), respectively. For BM-MSCs, conjugated antibodies against CD34, CD45, CD73, CD90, and CD105 (eBioscience, Langerwehe, Germany) were diluted in flow cytometry buffer, resulting in concentrations of 0.5 μg/100 μL, 0.06 μg/100 μL, 0.124 μg/100 μL, 1 μg/100 μL, and 1 μg/100 μL, respectively. For HUVECs, conjugated antibodies against CD31 (Becton Dickinson, Germany), CD45 (eBioscience, Langerwehe, Germany), and von Willebrand factor (Abcam, Cambridge, UK) were diluted in flow cytometry buffer. Following a 30 min incubation period at 4 °C, the cells were centrifuged and resuspended in 300 μL flow cytometry buffer for measurements. For each donor, a minimum of 10,000 events were carried out using a FACS Canto II cytometer (BD Bioscience, Heidelberg, Germany).

### 4.3. Angiogenesis Without Added Hydrogel

To obtain a supporting feeder layer, 40,000 BM-MSCs in SCM were seeded in a 24-well plate. After overnight incubation, the same amount of HUVECs was seeded in EGM-2 on top of the BM-MSC layer. Three different approaches with (i) 2.5 ng/mL figure (Lonza, Germany) per well, (ii) 9.6 µg/mL suramin (Merck, Darmstadt, Germany) per well, and (iii) untreated control were investigated in duplicate. Therefore, VEGF as a stimulator of angiogenesis [46] and suramin as an inhibitor of angiogenesis [47] were tested. Control wells with monocultures of either HUVECs or BM-MSCs were prepared. Four hours after HUVEC seeding, stimulating or inhibiting factors were added. The medium and VEGF/suramin supplementation were renewed every other day.

### 4.4. Immunofluorescence Staining and Fluorescence Microscopy

Cells were fixed with 4% p-FA (Morphisto, Offenbach, Germany) for 60 min. The fixed samples were blocked with 3% BSA (Carl Roth, Karlsruhe, Germany) for 60 min and washed with PBS. Then, CD31-antibody solution (mouse anti-human anti-CD31 (Abcam, UK), 1:400 in 1% BSA) was added to each well and the samples were incubated overnight at 37 °C in a humidified chamber. The samples were washed with PBS before secondary antibody solution (goat anti-mouse labelled with Alexa Fluor 555 (Thermo Fisher Scientifics, Braunschweig, Germany)) was applied and washed off the samples. Samples were treated with DAPI solution (1 µg/mL) for 10 min at RT, then washed and stored until image acquisition at the microscope. For qualitative analysis, five to seven images were taken per well with a 100×/1.40NA Oil immersion objective with the fluorescence microscope Leica CTR6000 (Leica, Wetzlar, Germany).

### 4.5. Quantification

Quantification was performed using the Angiogenesis Analyzer for ImageJ as previously described [32]. Thereby, the images were converted into 8-bit binary images and processed for noise reduction before quantification. The program processes the capillary-like structures by determining junction points and sections in between, called segments, as well as branches with open ends. This enables the measurement of characteristics such as number of branches/segments/junctions, total branch/segment length, and total mesh area, which can be compared between different samples.

### 4.6. Hydrogel Synthesis

Collagen gels were made from VitroCol^®^ human type I collagen G solution (3 mg/mL collagen I and III in 12 mM HCl, Advanced Biomatrix, Carlsbad, CA, USA). For preparation of collagen gels, 80% of the final gel suspension volume of collagen was mixed with 10% of the final gel suspension volume of 10× Dulbecco’s Modified Eagle’s Medium (DMEM, 45 g/L D-Glucose) and subsequently neutralized with 1 M natrium hydroxide. Then, 10% of the final gel suspension volume of EGM-2 was added with a BM-MSC and HUVEC cell suspension. The gels were incubated at 37 °C for around 1.5 h for the polymerization before covering them with medium.

Fibrin gels were synthetized according to a previously published protocol [26]. Fibrinogen and thrombin were obtained from TISSEEL fibrin sealant by Baxter International by emptying the syringe chambers separately. Fibrinogen was used in a concentration of 6.2 mg/mL in incomplete GBSH5 buffer (containing 0.37 g/L KCl, 0.2 g/L MgCl_2_ in 6H_2_O, 0.15 g/L MgSO_4_ in 7H_2_O, 7.00 g/L NaCl, 0.12 g/L Na_2_HPO_4_, 1.19 g/L HEPES) before use. When preparing fibrin-based hydrogels, a fibrinogen buffer suspension was prepared according to Table 1. For gels with cells, the cells were dissolved in the needed GBSH5 with DMEM 4.5 g/L D-glucose. Thrombin and fibrinogen buffer suspension were mixed quickly before gels were incubated at 37 °C for about 15 min until the gel was polymerized completely and then covered with EGM-2.

Usage of humane platelet lysate gels as three-dimensional matrix for cell growth was generated as previously published [26]. Gels were prepared by mixing a 1:1 ratio of HPL and EGM-2 and directly pipetting the solution into well plates. The fibrinogen from the plasma fraction of the platelet units’ coagulation cascade that was previously inhibited by chelation of calcium ions with citrate then started to coagulate the medium due to the addition of excess calcium ions via EGM-2. Gel formation occurred after a maximum of 1 h at 37 °C, and the gels were covered with EGM-2 after polymerization occurred. For angiogenesis experiments with hydrogels, the needed cell number was resuspended in the EGM-2 part of the gel before HPL addition. Hydrogels were prepared in 48-well plates with a volume of 450 µL each.

### 4.7. Scanning Electron Microscopy

The visual impression on the ultrastructure of the three different hydrogel types, visualized by scanning electron microscopy (SEM), was performed in triplicate for the time points of day 1 and day 10. The samples were fixed in 3% glutaraldehyde, rinsed in PBS, dehydrated in a graded ethanol series, critical point dried, mounted on SEM stubs, sputtered with 10 nm gold/palladium, and imaged using a Quattro S scanning electron microscope (Thermo Fisher, Germany).

### 4.8. Nanoindentation

To determine the mechanical properties and measure the stability of the hydrogels over the period of the experiment, nanoindentation was performed using a Piuma nanoindenter (Optics 11, Amsterdam, The Netherlands). Hydrogels were prepared in triplicate for each time point. To ease removal, a sterile glass platelet covered the bottom of the well. On day 1 or 10, the supernatant was removed, and the hydrogels were stored in PBS at 4 °C. For measurements, the samples were transferred to a Petri dish and fixed with gelatin. The nanoindenter was fitted with a 0.49 N m^−1^ probe with a 50.5 μm spherical tip radius. For each hydrogel, four different points in the middle of the gel, separated by 500 μm, were measured. Each point was measured three times. Indentation depth was set to 10,000 nm, and the indentation speed was 2000 nm/s. The values of Young’s modulus in Pascal (Pa) were determined automatically applying the Hertzian model with 100% fit for Pmax.

### 4.9. 3D Angiogenesis

For 3D angiogenesis experiments, 0.5 × 10^6^ cells/mL gel per cell type were seeded in the three different hydrogels during the polymerization process. All cells were trypsinized, counted, and resuspended in the required amount of EGM-2 or GBSH5 buffer, depending on the gel type. The collagen, fibrin, and HPL hydrogels were prepared in the 24-well plate format. The same three conditions that were tested during the angiogenesis without hydrogel were compared in the angiogenesis experiments with hydrogels. The corresponding concentration of VEGF/suramin was added for four hours of incubation after the polymerization of the gel. The culture medium was routinely refreshed every other day, ensuring consistent maintenance of both VEGF and suramin concentrations throughout the duration of the experiment. After 1, 7, and 10 days, the experiment was stopped by 2% p-FA fixation for 1 h at RT. All samples were then covered by PBS and stored at 4 °C for at least 24 h and until they were processed.

The experiment was repeated with three different MSC donors in each iteration. To assess the angiogenic potential of the gels without the assistance of MSCs, control experiments were conducted by seeding a monoculture of HUVECs at a density of 0.5 × 10⁶ cells/mL gel in each of the three hydrogel types, using the same culture conditions as in the co-culture experiments.

### 4.10. Immunofluorescence Staining

Immunofluorescence staining of angiogenesis experiments with hydrogels was carried out according to the staining for the angiogenesis experiments without hydrogels with some adaptations. To facilitate 2-photon microscopy, Alexa Fluor 594 (Thermo Fisher Scientific, Germany) was used as the secondary antibody. For staining, 200 µL of antibody solution were used, and to ensure thorough washing, washing steps were performed overnight at 4 °C. For DAPI, 200 µL with a concentration of 0.5 µg/mL were used. In addition to five to seven images taken at 100× magnification, two images with 50× and 200× magnification, respectively, were created for each well.

### 4.11. 2-Photon-Microscopy

To assess the three-dimensional nature of the cell growth and structures formed in the hydrogels, 2-photon microscopy was performed using Leica Stellaris 8 Dive Falcon multiphoton microscope equipped with a Spectra Physics Insight X3 Dual excitation laser and an HC IRAPO L 25x/1.00 W motCORR objective. For 2-photon microscopy, the samples that were previously stained against CD31 and with DAPI for fluorescence microscope imaging were used. Two detection windows of 4Tune HyD RLD detectors were arranged accordingly: 450–500 nm (DAPI), 620–670 nm (CD31/AF555).

### 4.12. Transmission Electron Microscopy

Samples were fixed with 3% glutaraldehyde in a phosphate buffer, post-fixed with 1% osmium tetroxide, dehydrated through an ethanol series, and embedded in epoxy resin. Ultrathin sections were stained with 0.5% uranyl acetate and 1% lead citrate and imaged using a Zeiss Leo 906 transmission electron microscope.

### 4.13. Statistical Analysis

For graphs and statistical analyses, GraphPad Prism 10.0.0 was used Two-way ANOVA was applied (*p* ** ≤ 0.005, *p* * ≤ 0.01) with a Tukey post hoc test. Mean and standard deviations (SDs) are shown in graphs.

## Figures and Tables

**Table 1:** Composition of fibrin-based hydrogels.

Component	µL
Fibrinogen (6.2 mg/mL)	288.98
CaCl_2_ (50 mM)	16.04
GBSH_5_	6.42
Tranexamic acid (100 mg/mL)	6.42
∑ Fibrogen-buffer suspension	317.86
Thrombin (10 U)	32.1

## Data Availability

The original contributions presented in this study are included in the article/Supplementary Materials. Further inquiries can be directed to the corresponding author.

## Supplementary Materials

The following supporting information can be downloaded at: https://www.mdpi.com/article/10.3390/gels10120820/s1, Figure S1: Angiogenesis in hydrogels—representative immunofluorescence pictures on days 1, 7, and 10. Experiments were repeated with three different MSC donors. CD31 is shown in red and cell nuclei in blue (DAPI). Images were taken at 100× magnification; scale bar: 200 μm; Figure S2: Angiogenesis in hydrogels—representative immunofluorescence pictures on days 1, 7, and 10. Experiments were repeated with three different MSC donors. CD31 is shown in red and cell nuclei in blue (DAPI). Images were taken at 100× magnification; scale bar: 200 μm; Figure S3: Angiogenesis in hydrogels—representative immunofluorescence pictures on days 1, 7, and 10. Experiments were repeated with three different MSC donors. CD31 is shown in red and cell nuclei in blue (DAPI). Images were taken at 100× magnification; scale bar: 200 μm.
