# Supplementary figures and images for: Various Hydrogel Types as a Potential In Vitro Angiogenesis Model
Source: Gels. 2024 Dec 12;10(12):820. doi: 10.3390/gels10120820 (PMC11675469; doi:10.3390/gels10120820)

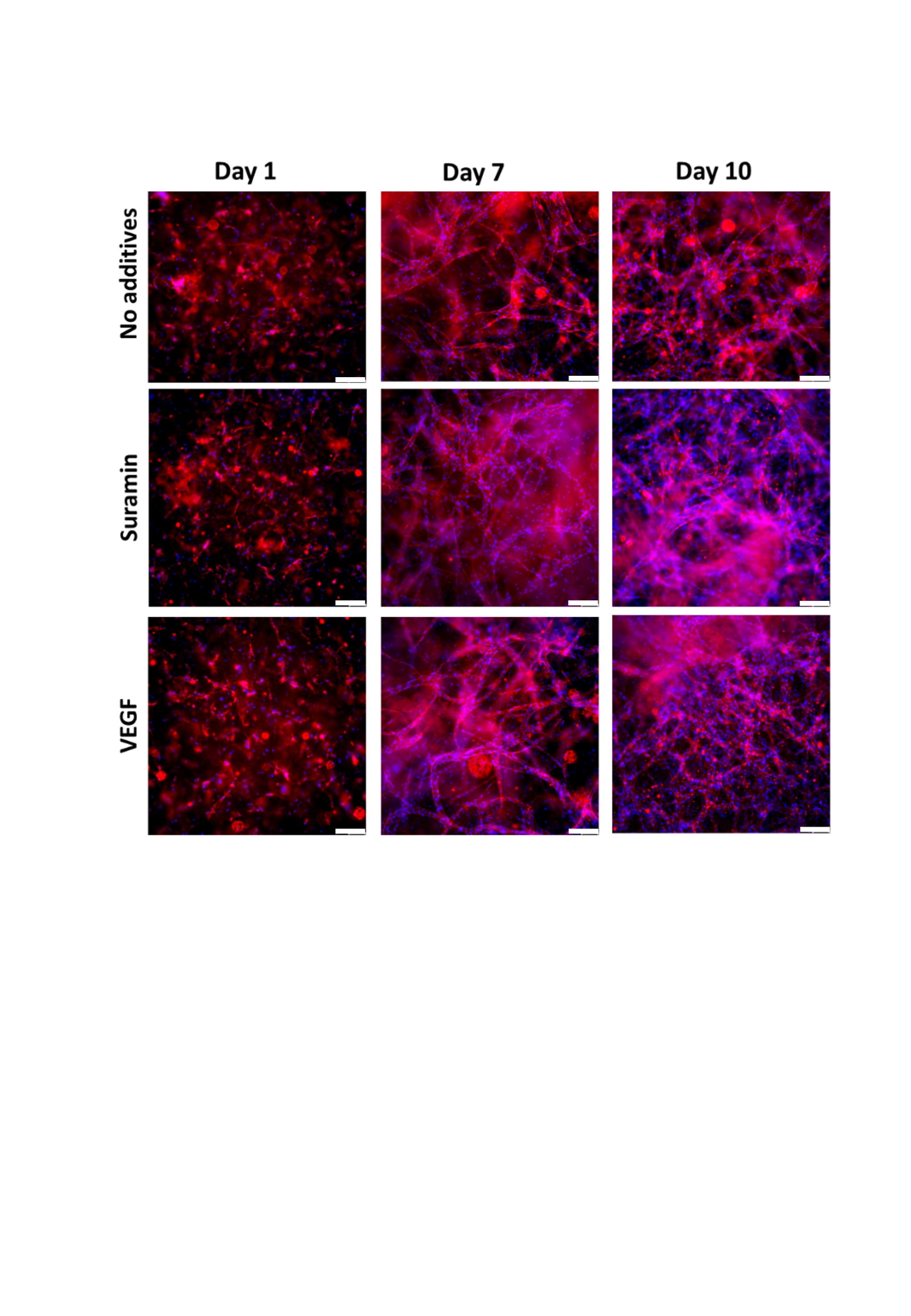

Supplement: Supplementary file 1 [file gels-10-00820-s001.zip › SuppData/Supp2-HPL-MajRev1_FINAL.tiff]

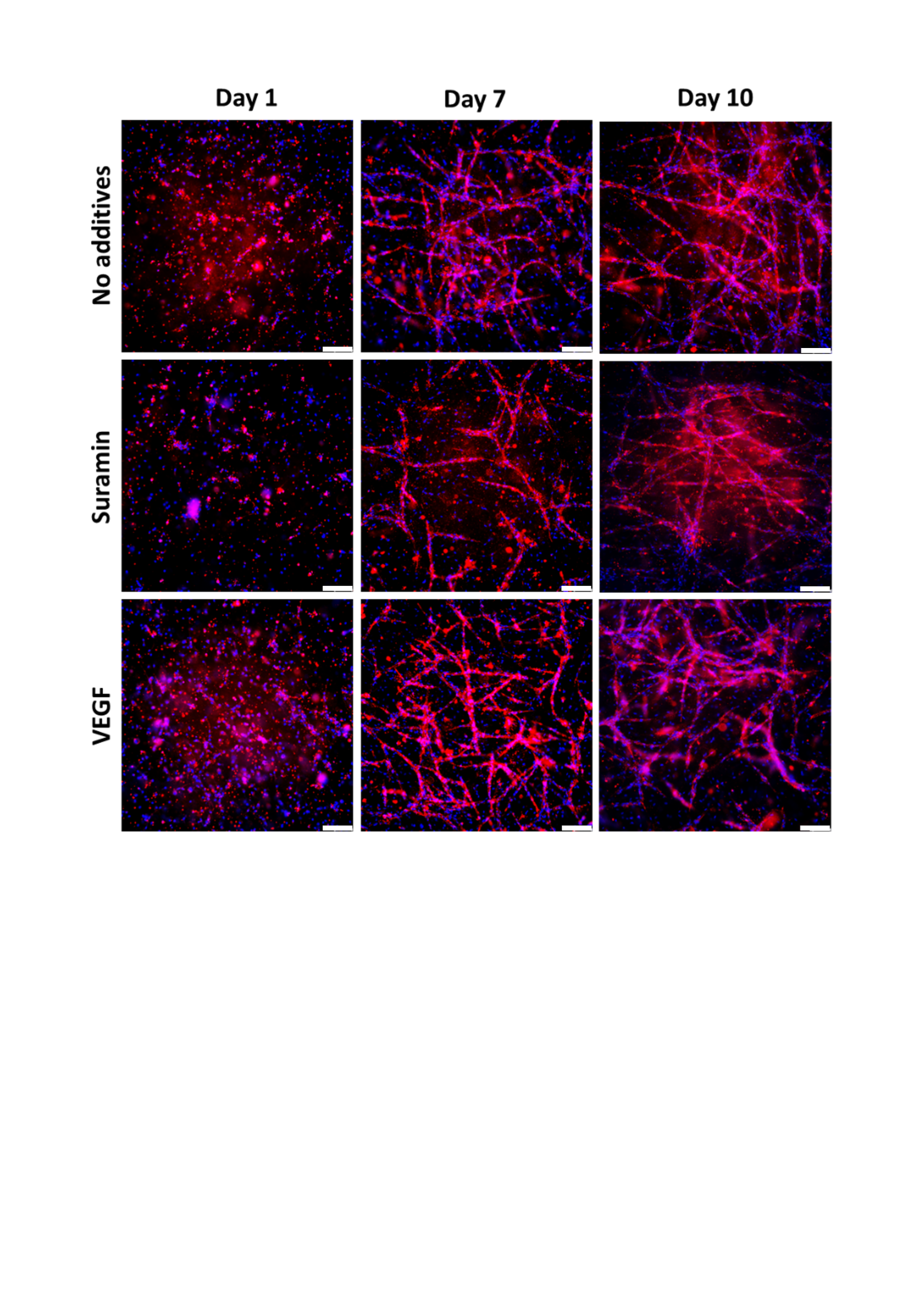

Supplement: Supplementary file 1 [file gels-10-00820-s001.zip › SuppData/Supp3-Coll-MajRev1_FINAL.tiff]

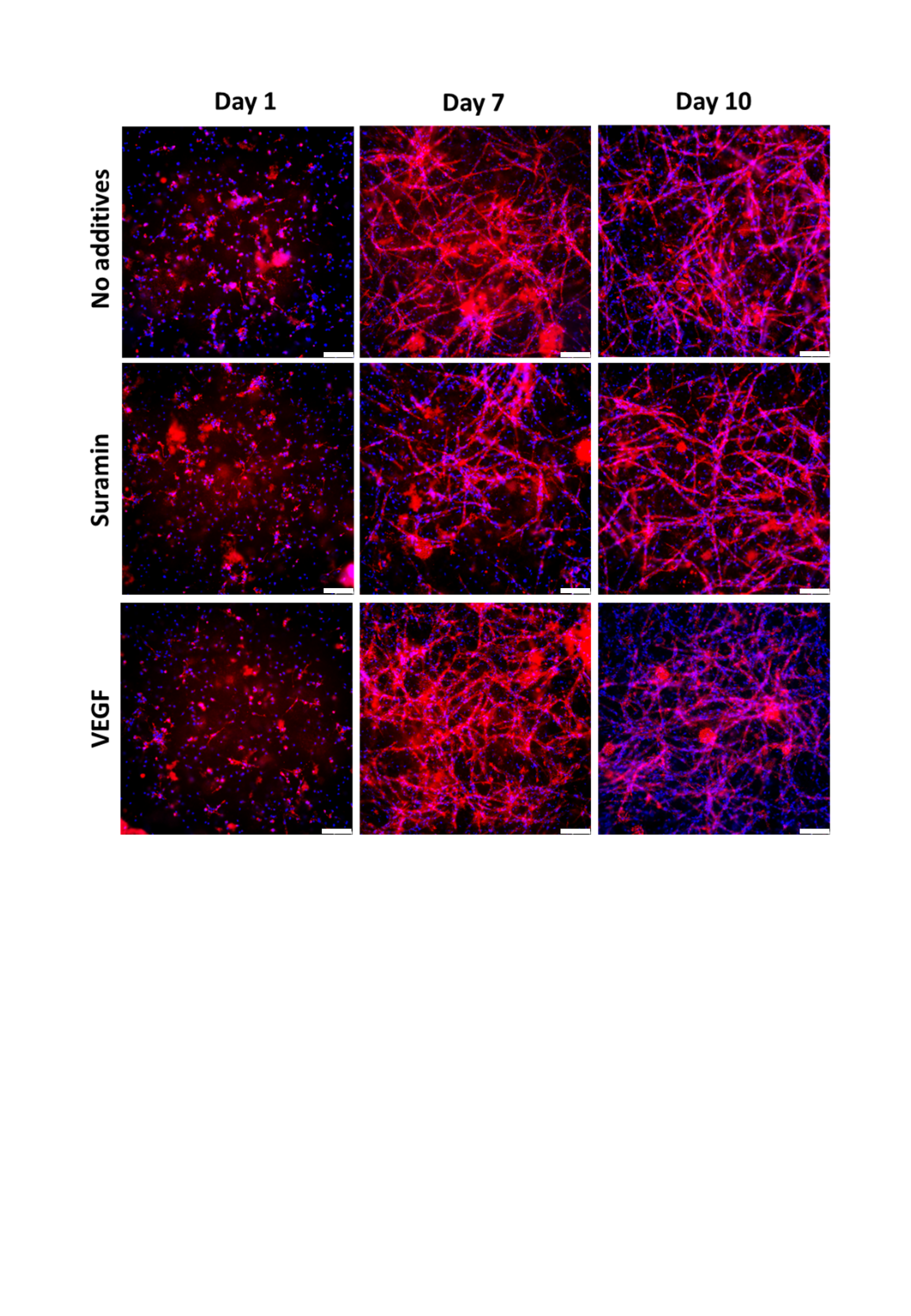

Supplement: Supplementary file 1 [file gels-10-00820-s001.zip › SuppData/Supp1-Fibrin-MajRev1_FINAL.tiff]
